# Insights into the Dichotomous Regulation of SOD2 in Cancer

**DOI:** 10.3390/antiox6040086

**Published:** 2017-11-03

**Authors:** Yeon Soo Kim, Piyushi Gupta Vallur, Rébécca Phaëton, Karthikeyan Mythreye, Nadine Hempel

**Affiliations:** 1 Department of Pharmacology, College of Medicine, Penn State University, Hershey, PA 17033, USA; ykim5@pennstatehealth.psu.edu (Y.S.K.); pvallur@pennstatehealth.psu.edu (P.G.V.); 2 Department of Obstetrics & Gynecology & Department of Microbiology and Immunology, College of Medicine, Penn State University, Hershey, PA 17033, USA; rphaeton@pennstatehealth.psu.edu; 3 Department of Chemistry and Biochemistry, University of South Carolina, Columbia, SC 29208, USA; KARTHIKE@mailbox.sc.edu

**Keywords:** superoxide dismutase, SOD2, MnSOD, SOD2 regulation, cancer

## Abstract

While loss of antioxidant expression and the resultant oxidant-dependent damage to cellular macromolecules is key to tumorigenesis, it has become evident that effective oxidant scavenging is conversely necessary for successful metastatic spread. This dichotomous role of antioxidant enzymes in cancer highlights their context-dependent regulation during different stages of tumor development. A prominent example of an antioxidant enzyme with such a dichotomous role and regulation is the mitochondria-localized manganese superoxide dismutase SOD2 (MnSOD). SOD2 has both tumor suppressive and promoting functions, which are primarily related to its role as a mitochondrial superoxide scavenger and H_2_O_2_ regulator. However, unlike true tumor suppressor- or onco-genes, the *SOD2* gene is not frequently lost, or rarely mutated or amplified in cancer. This allows *SOD2* to be either repressed or activated contingent on context-dependent stimuli, leading to its dichotomous function in cancer. Here, we describe some of the mechanisms that underlie SOD2 regulation in tumor cells. While much is known about the transcriptional regulation of the *SOD2* gene, including downregulation by epigenetics and activation by stress response transcription factors, further research is required to understand the post-translational modifications that regulate SOD2 activity in cancer cells. Moreover, future work examining the spatio-temporal nature of SOD2 regulation in the context of changing tumor microenvironments is necessary to allows us to better design oxidant- or antioxidant-based therapeutic strategies that target the adaptable antioxidant repertoire of tumor cells.

## 1. Introduction

**Figure 1:**
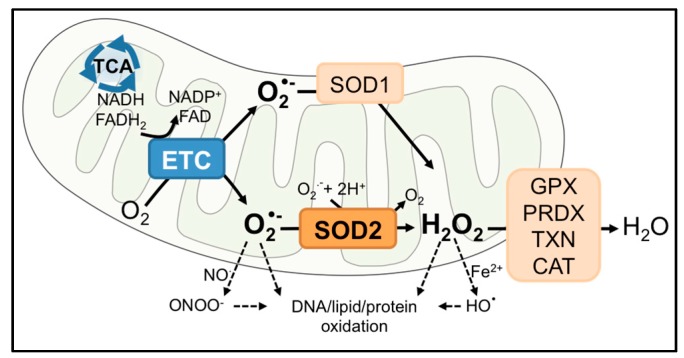
Function of mitochondrial SOD2. SOD2 is localized in the mitochondrial matrix to catalyze the dismutation of O_2_•^−^ to H_2_O_2_. H_2_O_2_ is further metabolized by catalase (CAT) in the peroxisomes or by the glutathione peroxidase (GPX), peroxiredoxin (PRDX)/thioredoxin (TXN) system, isoforms of which are found in the mitochondria and the cytosol. O_2_•^−^ and H_2_O_2_ can react to form other oxidants, such as peroxynitrite (ONOO^−^) or hydroxyl radical (HO•). Depending on the reactivity or amount of the oxidant, subsequent oxidation of macromolecules can lead to variable degrees of cellular outcomes, including changes in redox signaling (e.g., oxidation and inactivation of phosphatase) or irreversible changes (DNA damage).

In aerobic organisms, cellular respiration is essential for energy production. To generate the proton gradient necessary for Adenosine Triphosphate (ATP) synthesis at complex V in the mitochondrial electron transport chain (ETC), electrons pass from reducing equivalents, such as nicotinamide adenine dinucleotide (NADH) and Flavin adenine dinucleotide (FADH_2_), to ETC complexes, where oxygen (O_2_) serves as a final electron acceptor at complex IV. However, leakage of electrons at complexes I and III can results in partial reduction of O_2_ to superoxide anion free radical (O_2_•^−^), a short-lived oxidant that can either directly react with biomolecules or be dismutated to H_2_O_2_ by superoxide dismutases (SOD) [1]. Therefore, the flow of O_2_ into mitochondria not only promotes continuous ATP generation, but also makes the organelle a major source of intracellular reactive oxygen species (ROS; Figure 1). An uncontrolled buildup of ROS can be deleterious to mitochondrial function and the cell. Commonly, this results in oxidation and inactivation of metabolic and electron transport chain enzymes, mitochondrial DNA damage and the release of cytochrome c to initiate apoptosis [2,3,4,5]. In contrast, sub-lethal increases in mitochondrial oxidant generation can lead to ROS-mediated signaling that regulates diverse cellular processes, as discussed further in Section 3 below.

Since O_2_•^−^ generation occurs even during normal respiration, aerobic organisms have acquired defense mechanisms to protect mitochondria from oxidative damage and to maintain redox homeostasis. An imbalance of this homeostasis is associated with numerous pathologies, including cancer. We have come to appreciate that the role of oxidants and their counteracting antioxidant enzymes in tumorigenesis and metastatic progression is complex. On the one hand, a loss of antioxidant enzyme expression can lead to the initiation of tumorigenesis through the buildup of oxidants that damage macromolecules, such as DNA; while on the other hand, increased oxidant scavenging provides tumor cells with survival advantages in situations of redox stress, including those associated with metastatic progression and chemo-and radio-therapy. In both scenarios, a change in the balance of different oxidant species can also have consequences on their specific actions as signaling molecules, as described in more detail below. In the present review, we will discuss the dichotomous role and regulation of mitochondrial superoxide dismutase (SOD2) in this context.

## 2. SOD2

The cell contains a sophisticated antioxidant system that is compartmentalized to effectively eliminate ROS within sites of their generation. Mitochondria are particularly vulnerable to oxidative damage due to their O_2_-dependent metabolic functions and the presence of redox-sensitive enzymes [3,4]. As a result, mitochondria contain antioxidant enzymes in both the intermembrane space and matrix, including superoxide dismutases 1 and 2 (SOD1 and 2), peroxiredoxin (PRDX3), thioredoxin (TXN2) and glutathione peroxidase (GPX1). This review focuses on manganese superoxide dismutase 2 (SOD2, or MnSOD), which converts O_2_•^−^ to hydrogen peroxide (H_2_O_2_) and molecular oxygen (Figure 1). Of the three members of the SOD family, SOD1 and SOD2 are found within the mitochondria, while SOD3 is primarily localized extracellularly. SOD1 occupies both the cytoplasm and the mitochondrial intermembrane space. SOD2 is primarily located in the mitochondrial matrix, a site of O_2_•^−^ production by complexes I and III of the ETC. The SOD enzymes also differ in the composition of metal cofactors essential for their dismutase activity. SOD1 is a homodimer equipped with Cu/Zn, while SOD2 is a homotetramer with a manganese ion (Mn^2+^/Mn^3+^) cofactor in each subunit. SOD2 is a nuclear-encoded enzyme and translocates into the mitochondrial matrix post-translationally. In the matrix, incorporation of Mn into the catalytic site of SOD2 makes the enzyme competent to perform dismutase activity [6,7]. Although both enzymes scavenge O_2_•^−^ in a similar manner, the antioxidant functions of SOD2 in the mitochondrial matrix cannot be replaced by the presence of SOD1 in the intermembrane space and cytosol, as SOD2 knockout mice exhibit neonatal lethality even with SOD1 overexpression [8]. This highlights the indispensable role of SOD2 in the mitochondrial matrix.

## 3. Dichotomous Role of SOD2 in Cancer

The relative amount and reactivity of oxidant species arising from the mitochondria can have various consequences on cellular functions. These range from redox signaling, by reversible oxidation of phosphatases and transcription factors, to DNA and protein damage, which can result in deleterious consequences such as mutations and cell death. 

**Figure 2:**
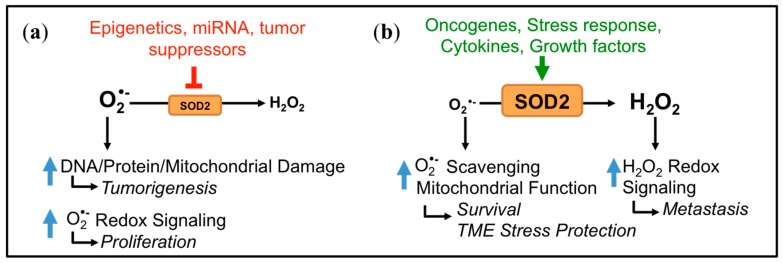
The dichotomous role and regulation of SOD2 in cancer. SOD2 changes the redox status of mitochondria, whereby negative regulation of SOD2 can result in O_2_•^−^-driven tumorigenesis and proliferation, supporting a tumor-suppressive function for SOD2 (**a**). Conversely, activation of SOD2 enhances O_2_•^−^ scavenging to adapt to stress associated with tumor progression and can drive H_2_O_2_-mediated signaling, resulting in the role of SOD2 as a tumor promoter (**b**); TME: tumor microenvironment).

The function of SOD2 as either a tumor suppressor or promoter is intimately linked to its function as a regulator of mitochondrial oxidants, specifically O_2_•^−^ and H_2_O_2_ (Figure 1 and Figure 2). Due to its cytoprotective function of scavenging harmful O_2_•^−^ at the major cellular metabolic hub, SOD2 was traditionally considered a tumor suppressor. This was based on observations that SOD2 expression is decreased in some tumors compared to their normal tissue counterparts and that forced overexpression of SOD2 significantly delayed tumor cell growth in nude mice xenograft studies of several tumor types [9,10]. A reduction or loss of SOD2 expression is thought to mediate transformation and tumorigenesis by increasing ROS-mediated DNA damage, as a consequence of the accumulation of O_2_•^−^ and oxidants generated from O_2_•^−^, such as ONOO^−^, H_2_O_2_ and HO• radical [11,12,13] (Figure 1 and Figure 2a). In addition, changes in sublethal O_2_•^−^ levels have been associated with different stages of the cell cycle. Increases in O_2_•^−^ are linked with mitosis, whereas H_2_O_2_ levels rise during the G0/G1 phase [14]. Changes in SOD2 activity have similarly been reported during different stages of the cell cycle [15], and a decrease in SOD2 expression may therefore stimulate cell cycle progression by increasing O_2_•^−^ levels [16,17]. The above suggest that a loss of SOD2 expression may be a phenotype of tumor initiation and that the tumor suppressor role of SOD2 is primarily linked to its role as a O_2_•^−^ scavenger during tumorigenesis (Figure 2a). 

Interestingly, it was subsequently shown that changes in SOD2 expression and activity are tumor type-dependent, with some cancers displaying increased SOD2 levels compared to their normal tissues of origin [18,19,20] (Table 1). This has been confirmed by the analysis of large expression datasets [21]. An additional important observation is the dichotomous role and regulation of SOD2 during tumor progression. As mentioned above, a loss of SOD2 expression is frequently observed during tumor initiation, such as early tumor lesions and non-metastatic cancer cell lines. However, during metastatic progression, SOD2 levels appear to increase, as seen in metastatic tumor lesions and highly aggressive tumor cell lines [19,21,22]. Several studies have shown that elevated antioxidant enzyme expression is a necessary survival adaptation during tumor progression. This enables cells to cope with increased cellular and extracellular redox stress, such as that associated with matrix detachment [23,24,25]. SOD2 expression similarly provides a protective advantage to tumor cell mitochondria through SOD2’s canonical role as a O_2_•^−^ scavenger (Figure 2b). In addition, we and others have demonstrated that increased SOD2 activity also shifts the redox balance towards a higher cellular steady-state H_2_O_2_ status in tumor cells [19,26,27,28,29]. A moderate sub-lethal H_2_O_2_ increase driven by SOD2 expression can elicit oxidation and inactivation of phosphatases, resulting in enhanced redox signaling. For example, oxidation of the phosphatases PTEN (phosphatase and tensin homolog) and PTP-N12 (Protein Tyrosine Phosphatase N12) increases in response to SOD2 expression [26,27,28,30]. This leads to enhanced activation of Akt and p130cas/Rac1, respectively, signaling pathways that regulate migration, invasion and angiogenesis pathways during tumor progression [26,27,28,30]. Hence, increased SOD2 expression likely plays a dual role in aiding tumor progression. First, SOD2 preserves mitochondrial function through canonical O_2_•^−^ scavenging, and second, SOD2 shifts the cellular redox landscape to elicit H_2_O_2_-dependent redox signaling (Figure 2b).

For more extensive reviews on the dichotomous function of SOD2 in cancer and mitochondrial redox signaling, we refer the reader to the following reviews [21,31,32,33,34,35]. Below, we focus on the regulatory mechanisms governing either activation or repression of SOD2 in cancer, ranging from transcription to posttranslational modifications. Examples of these and how different tumor types and stages of cancer can influence SOD2 regulation are provided (Table 1). We acknowledge that this is not a complete list and apologize for any omissions due to space constraints.

## 4. Transcriptional Regulation of *SOD2*

**Figure 3:**
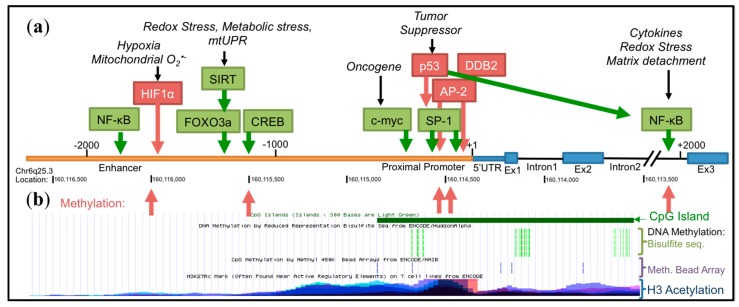
The *SOD2* promoter. (**a**) Transcription factors that activate *SOD2* transcription are shown in green, while repressors are represented in red. The promoter contains a proximal promoter and 5′ and intronic enhancer regions. (**b**) Red arrows show areas of demonstrated DNA methylation. Corresponding University of California Santa Cruz (UCSC) Genome Browser data (Human Assembly GRCh37/hg19, Chr 6q25.3; note: *SOD2* is located on the reverse strand) demonstrate the location of the CpG island spanning the proximal promoter to Intron 2 and confirmed DNA methylation marks from bisulfite sequencing and methylation bead arrays. Areas of acetylated H3 histone association are also shown.

As one of the essential players of the intracellular antioxidant system, SOD2 is expressed constitutively in most tissues to protect mitochondria from a steady production of O_2_•^−^ by the ETC during normal respiration. It has become apparent that SOD2 transcription is dynamically regulated based on its context-dependent role in cancer. Unlike true tumor suppressors or oncogenes, the SOD2 gene, located on chromosome 6q25.3, is not frequently lost, or rarely mutated or amplified in cancer. Although chromosome 6q instability and loss of heterozygosity (LOH) have been reported in melanoma [64] and associated with low SOD2 expression during the process of SV40 fibroblast transformation [80], SOD2’s role as a tumor suppressor is most commonly associated with SOD2 expression decreases through *SOD2* epigenetic regulation or inhibition of tumor associated signaling that affect the basal and enhancer transcriptional machinery. In contrast, high SOD2 expression is most commonly associated with transcriptional upregulation in response to intra- and extra-cellular stimuli, including those associated with redox stress. The *SOD2* gene contains three main regulatory regions (5′ upstream enhancer region, proximal promoter, second intronic enhancer region), which are responsible for binding transcription factors involved in *SOD2* transcriptional activation or repression (Figure 3). Below, we highlight the major transcriptional mechanisms responsible for both inhibitory and activating *SOD2* gene regulation in the context of cancer.

### 4.1. Basal Transcription

The proximal promoter of *SOD2* does not have a TATA or CAAT box, but is instead enriched with CG-repeats that contain binding sites for specificity protein 1 (Sp1) and activator protein 2 (AP-2) [81]. Sp1 binding to the promoter activates *SOD2* transcription, whereas AP-2 suppresses it by competing for Sp1 at its binding site or limiting Sp1 availability by forming a complex with Sp1 [81,82]. Differential expression of SOD2 in cancer cells is in part due to the changes in relative abundance of Sp1 and AP-2 [83]. For example, hypermethylation of the *AP-2* gene decreases AP-2 expression in aggressive breast cancer cells [84], where high SOD2 expression is often observed relative to non-invasive breast cancer cells [50]. Conversely, mutations found in the *SOD2* gene promoter influence the binding pattern of AP-2 in colorectal cancer cells and downregulate SOD2 expression [56]. Altogether, Sp1 and AP-2 modulate transcription of *SOD2* in the proximal promoter region, and their antagonistic functions fine-tune the basal expression level of SOD2. The binding of these can be influenced by other transcription factors that may be altered in cancer to either negatively or positively regulate the *SOD2* promoter, damaged DNA binding 2 (DDB2) being an example of negatively influencing *SOD2* basal transcription. A binding site for DDB2 has been identified at position −230, and it is thought that DDB2 represses *SOD2* transcription in breast cancer cells through interaction with AP-2, inhibiting the recruitment of Sp1 to the promoter [49]. The inverse correlation between SOD2 and DDB2 expression in breast cancer patient-derived samples further establishes DDB2 as a negative transcriptional regulator [50].

### 4.2. Epigenetic Regulation 

Epigenetic regulation of *SOD2* transcription is primarily associated with decreased SOD2 expression in cancer. Several studies have reported epigenetic regulation of the *SOD2* gene in both transformed and cancer cells [65,66,69,85], including *SOD2* promoter methylation and association with repressive histone modifications. The long repertoire of CG dinucleotide repeats spanning the *SOD2* promoter and extending into the second intronic region (Figure 3) is highly sensitive to methylation. One of the proposed mechanisms of SOD2 downregulation in early stages of cancer involves aberrant patterns of cytosine methylation. The Domann group has extensively investigated the epigenetic regulation of SODs and showed that SV40-transformed human fibroblasts with low SOD2 expression have hypermethylated cytosine residues in the second intron [12]. The *SOD2* promoter has been shown to have a higher frequency of methylation in a number of cancer cell lines including multiple myeloma, breast and pancreatic cell lines with low SOD2 expression [52,65,66,69]. Methylation of CG repeats can occur within the AP-2 and Sp1 binding sites in the *SOD2* proximal promoter, as well as enhancer regions, which interfere with the binding of transcriptional activators at these sites (for a comprehensive review on *SOD2* epigenetic regulation, see [86]). In addition to DNA methylation, transcriptional suppression of *SOD2* in breast cancer cells is associated with repressive histone marks (decreased H3K4me2 and H3K9ac) and condensed chromatin structure in the regulatory regions [51]. Interestingly, transcriptional activating H3 histone marks are associated with increased SOD2 expression in aggressive breast cancer. This results in increased binding of nuclear factor κB (NF-κB) to the intronic enhancer element, which is an activator of *SOD2* transcription, as described in more detail below [50]. These results suggest that dynamic epigenetic alterations in the *SOD2* gene may contribute to both decreased expression in cancer development and enhanced SOD2 levels during tumor progression. Future studies examining the role of altered tumor metabolism and different tumor metastatic niches on epigenetic regulation should further shed light on the influence of context-dependent epigenetic regulation of *SOD2*.

### 4.3. Influence of Oncogenes and Tumor Suppressors

The underlying genetic landscape of tumors, including expression of oncogenes or tumor suppressors, may also impact *SOD2* transcription. An example is the regulation of *SOD2* by c-myc in tongue squamous cell carcinoma. A recent study has revealed a potential c-myc binding site (5′-GGGCACGTCGT-3′) located at position −612, using chromatin immuno-precipitation (ChIP) and *SOD2* promoter activity assays [78]. MYC proto-oncogene (c-myc)-dependent increases in SOD2 expression lead to a more migratory and invasive phenotype, and enhance stem cell properties of tongue squamous cell carcinoma cells [78]. 

Much focus has been placed on the role of *SOD2* regulation by the DNA damage response protein and tumor suppressor p53, following observations that p53 status negatively correlates with SOD2 expression. Mutations in the tumor protein p53 (*TP53*) gene are among the most common observed in cancer [87], and the absence of functional p53 most often inversely correlates with SOD2 expression [88]. For example, knock-out of p53 increases SOD2 expression in transformed fibroblasts, while forced expression of p53 in HeLa cells decreases SOD2 expression [89]. Conversely, it has also been shown that p53 can induce SOD2 expression and that the lack of a similar induction of catalase results in an imbalance of H_2_O_2_ detoxification and increases in H_2_O_2_-dependent apoptosis [90]. 

p53 binding sites have been identified in the 5’ upstream region of the *SOD2* gene [88,90]; however, it appears that p53 regulates *SOD2* transcription primarily through its interaction with other transcription factors rather than direct binding to the predicted DNA region. p53 exerts its inhibitory action by preventing binding of Sp1 to the proximal promoter. Extensive work by the St. Clair group has delineated the role of p53 in *SOD2* regulation in cancer and importantly demonstrated potential mechanisms for the biphasic expression of SOD2 observed during tumor initiation and tumor progression, which are characterized by low SOD2 and high SOD2 expression, respectively. Using a skin carcinogenesis model, it was found that 7,12-dimethylbenz[a]anthracene (DMBA) and tetradecanoylphorbol-13-acetate (TPA) activate p53 during tumor initiation and lead to *SOD2* transcriptional repression, via inhibition of Sp1 binding to the proximal promoter [22,91]. As the tumors progress to squamous cell carcinomas, Sp1-dependent SOD2 expression increases as functional p53 is gradually lost [22,87]. The same group also demonstrated that the role of p53 on SOD2 regulation is p53 concentration dependent. While high p53 expression is suppressive, a low level p53 expression leads to increased NF-κB binding to the enhancer region of the *SOD2* gene and increased *SOD2* transcription [91,92]. The above studies demonstrate variable mechanisms by which p53 modulates SOD2 expression. Basal SOD2 expression may hence be increased in cancer types that display high levels of TP53 mutation, such as ovarian high-grade serous adenocarcinomas [93] or where TP53 activity is lost during tumor progression [87]. Interestingly, p53 can also regulate SOD2 activity independent of transcription. For example, TPA can drive p53 translocation to the mitochondria, where it can directly interact with and inhibit the activity of SOD2. This is an important step in apoptosis initiation, and the block of SOD2 by p53 drives mitochondrial oxidant overload, which is necessary in the apoptotic process [94]. Therefore, an increase in SOD2 expression and activity in response to p53 loss appears to be a cytoprotective mechanism of tumor cells.

### 4.4. Inducible Transcription

SOD2 increases during tumor progression have largely been linked to stress response pathways that positively regulate *SOD2* transcription, including Nuclear Factor, Erythroid 2 Like 2 (Nrf2) and NF-κB signaling. This is linked to intra- and extra-cellular redox stress in response to changing tumor states, an example being the surge in ROS associated with matrix detachments during metastasis, as well as extracellular signals from the tumor microenvironment, including growth factors and cytokines. Prominent examples of these are discussed below. However, there are also examples where dampening of these pathways leads to SOD2 repression in cancer. Enhancer regulatory domains for a number of transcription factors are found at two main locations in the *SOD2* gene, the 5′ upstream (~−1500 bp) and the intronic enhancer regions (~+1500, Figure 3). In the 5′ upstream enhancer region of the *SOD2* gene reside binding sites for activating protein (AP-1) [95], CAMP Responsive Element Binding Protein (CREB) [96], p53 [90], Nrf2 [97], Forkhead Box O3 (FoxO3a) [98], NF-κB and hypoxia inducible factor α (HIF-1α) [74], while the intronic enhancer is a primary site for NF-κB binding [99,100]. Binding of transcription factors at these sites often results in the interaction with the basal transcriptional machinery of the proximal promoter, such as Sp1, to regulate *SOD2* transcription. 

NF-κB is a well-known stress-responsive transcription factor activated upon various stimuli including cytokines [99], retinoids [101], irradiation [102] and matrix detachment [24]. Transcriptional regulation of *SOD2* by NF-κB was established in many studies using different experimental systems. A particular finding is that cells treated with tumor necrosis factor alpha (TNF-α) and interleukin 1 (IL-1) increase transcription of *SOD2* dramatically through NF-κB binding to the intronic enhancer region (5′-GGGAATACCC-3′) located in the second intron of the *SOD2* gene through interactions with CCAAT/Enhancer Binding Protein Beta (C/EBP-β) [99,100]. It was established that binding of NF-κB at the intronic enhancer region enhances the recruitment of the transcriptional activator Sp1 to the proximal *SOD2* promoter and is associated with histone hyperacetylation [50]. This has been described to underlie the high SOD2 expression observed in aggressive breast cancer cells [50]. Since the discovery of direct regulation of *SOD2* by NF-κB, it has been demonstrated that this stress response pathway is involved in anti-apoptosis [24], tumor metastasis [24] and radiation resistance [103]. TNF-α treated epithelial cancer cells defend against ROS-induced apoptosis via NF-κB-dependent SOD2 expression [104]. Not only do SOD2 levels increase in response to cytokines and growth factors, the Lu group has demonstrated that breast cancer cells elevate SOD2 expression under anchorage independence in an NF-κB-dependent manner [24]. This allows tumor cells to manage the enhanced oxidative stress associated with matrix detachment, which can result as a consequence of decreased glucose uptake in anchorage independence [105]. Overall, the role of NF-κB-dependent SOD2 expression is likely the primary mechanism for increased SOD2 expression in response to pro-survival cytokine stimulation and a mechanism for adaptations against cellular redox stress associated with tumor metastasis and the microenvironment.

The core sequence of the antioxidant responsive element (ARE), 5′-TGACnnnGC-3′, has been found in many genes encoding detoxifying enzymes such as NAD(P)H quinone oxidoreductase (NQO1) and γ-glutamylcysteine subunits (γ-GCS) [106]. Among many transcription factors that can recognize and bind to the ARE, Nrf2 has been shown to act as a master regulator of ARE-responsive antioxidant genes at the basal and induced conditions [107,108,109]. Oncogene-driven overexpression of Nrf2 contributes to tumorigenesis by activating the Nrf2-dependent antioxidant response program [110]. Nrf2 is bound to the regulatory protein Kelch ECH associating protein 1 (Keap1), which promotes ubiquitination and subsequent proteasomal degradation of Nrf2. In stressed conditions, modifications of Keap1 cysteine residues are thought to destabilize the interaction between Nrf2 and Keap1 [111,112] and/or impair Keap1-mediated Nrf2 ubiquitination [113], thus allowing nuclear translocation and transcriptional activity of Nrf2. Not surprisingly, gene mutations and epigenetic silencing of Keap1 have been associated with elevated levels of Nrf2 and chemoresistance in different cancers [68,114,115]. Interestingly, caveolin-1 can suppress the transcriptional activity of Nrf2 through direct binding [116], and furthermore, ectopic overexpression of caveolin-1 abrogates both Nrf2 and SOD2 expression in breast cancer, suggesting that the commonly observed loss of caveolin-1 in breast cancer is a driving factor for Nrf2-dependent SOD2 regulation [53]. Although direct Nrf2 binding to the ARE in the *SOD2* gene has not been shown so far, algorithm-based analysis predicted *SOD2* as a strong candidate of Nrf2 target genes [97]. Impaired SOD2 mRNA expression in Nrf2-disrupted mice after treatment with the chemo-protective drug 3H-1,2-dimethiole-3-thione (D3T) further provides evidence that *SOD2* could be a transcriptional target of Nrf2 [117]. The regulation of SOD2 by stress response transcription pathways such as Nrf2 is one example of how histological tumor subtypes can display differences in SOD2 expression. We demonstrated that SOD2 has a pro-metastatic role and is specifically elevated in ovarian clear cell carcinomas compared to other ovarian cancer subtypes [27]. This histological subtype displays high Nrf2 pathway upregulation due to enhanced presence of Keap1 mutations, and these Nrf2-high tumors display increased SOD2 expression [68]. 

FoxO3a is one of the Forkhead transcription factors in mammals and a well-known downstream target of phosphatidylinositol-4,5-bisphosphate 3-kinase (PI3K)/protein kinase B (PKB/Akt) signaling. Putative FoxO binding elements have been identified upstream of the SOD2 transcription start site, but the one at position −1249 is responsible for FoxO3a-dependent transcription of SOD2 in quiescent cells [98]. In pathological conditions, increased Akt activity promotes nuclear exclusion of FoxO3a through phosphorylation and downregulates SOD2 transcription [118,119]. Sirtuins (SIRT), a class of NAD^+^-dependent deacetylases, have been shown to regulate transcription of SOD2 indirectly via FoxO3a. SIRT2-mediated deacetylation of Foxo3a resulted in enhanced DNA binding and SOD2 expression under oxidative stress [120]. SIRT3 overexpression in colorectal cancer cells also decreased FoxO3a acetylation and increased FoxO3a binding to the *SOD2* promoter, whereas expression of a SIRT3 catalytic mutant did not affect the DNA binding ability of FoxO3a [121]. The FoxO3a regulation by SIRT3 has also recently been implicated as a necessary adaptation to the mitochondrial unfolded protein response (mtUPR), which occurs more frequently in tumor cells with accumulating mitochondrial DNA mutations. This mode of SOD2 regulation confers survival in response to the mtUPR [122]. Therefore, it appears that FoxO3a-mediated transcription of SOD2 is directly governed by the extent of activation of the PI3K-Akt signaling pathway and indirectly by an alteration in intracellular redox status and NAD^+^ availability through SIRTs. 

While the above represent major stress response pathways responsible for SOD2 regulation in cancer, other transcription factors have also been implicated with elevated SOD2 expression in response to redox stress, including the aryl hydrocarbon receptor nuclear translocator (ARNT) in acute myeloid leukemia (AML) [123]. Moreover, carcinogen-elicited stress drives transcription through various transcription factors of the *SOD2* promoter enhancer elements. For example, TPA treatment enhances CREB-1/Activating Transcription Factor 1 (ATF-1) to the CREB-like consensus binding sequence (5′-TGACGTCT-3′) at position −1258 to induce *SOD2* transcription [96].

HIF-1α-dependent inhibition of SOD2 transcription has been shown in renal clear cell carcinoma [74].The renal carcinoma a cell line RCC4, with constitutive expression of HIF-1, had low SOD2 expression, but this was reversed when the expression of von Hippel-Lindau tumor suppressor protein (VHL), an E3 ligase involved in HIF-1α degradation, was restored. ChIP analysis using a HIF-1α antibody revealed a hypoxia response element (HRE) in the region between positions −1426 and −1413 (5′-GCGTGGAGGTGCAC-3′) [74]. Given that mitochondrial O_2_•^−^ has been implicated in the activation of HIF-1α [124], decreased SOD2 expression by HIF-1α was proposed as a positive feedback mechanism for HIF-1α signaling in renal clear cell carcinoma [74].

### 4.5. Other Transcriptional Regulators

In stem cells, transcription of the *SOD2* gene has been shown to be Nanog and Oct4-dependent based on promoter activity assays and RNAi-mediated knockdown of the two pluripotency transcription factors [125]. Potential Nanog, Oct4, and Sox2 binding sites are located at the 5′ upstream of the SOD2 gene transcription start site, suggesting that these transcription factors may regulate SOD2 in a stem cell-like state [125]. Inhibiting mechanistic Target Of Rapamycin (mTOR) by rapamycin has also been demonstrated to increase SOD2 expression in self-renewing keratinocytes and confers radioprotective effects to these cells [126], as previously described for hematopoietic progenitor cells [127]. In addition, quiescent pancreatic cancer cells display increased SOD2 expression in a Mirk/Dyrk1B-dependent manner [128]. These studies point to heterogeneity in SOD2 regulation in different tumor cell subpopulations. The role and regulation of SOD2 in cancer initiating cells and quiescent cancer states, such as tumor dormancy require further investigation, and may reveal the mechanisms by which SOD2 confers survival advantages to these cells, which play an important role in their inherent chemo- and radio-resistance.

## 5. Post-Transcriptional Regulation of *SOD2*

The mRNA transcripts of SOD2 undergo various post-transcriptional modifications. One potential cause of dysregulation of SOD2 expression in cancer is mediated through miRNA. The 3′ untranslated region (UTR) of the SOD2 mRNA has the Alu-like element to which small antisense RNA binds in the cytoplasm and inhibits protein translation [129]. High expression of several miRNAs has been correlated with low SOD2 expression in cancer. miR-222 has been identified as a potential negative regulator of SOD2 expression through direct binding to the 3′ UTR in tongue squamous cell carcinoma [77]. Similarly, miR-382 decreases SOD2 following transforming growth factor 1 beta (TGF-1β) treatment [130]. Moreover, immunohistochemical staining of pancreatic ductal adenocarcinoma patients’ tissue samples revealed an inverse correlation between low SOD2 and high miR-301a expression [70]. 

In contrast to the above studies, a decrease in certain miRNAs has also been linked to high SOD2 expression in tumor cells. miR-17*, generated from the 3′ arm of its miRNA precursor (denoted by asterisk, miRNA*), downregulates the expression of antioxidant enzymes, including SOD2, glutathione peroxidase 2 (GPX2) and thioredoxin reductase 2 (TXNRD2), through binding to the putative miR-17* targeting regions of these genes [73]. Low miR-17* expression in the PC-3 prostate cancer cell line was associated with high SOD2 expression and other antioxidant enzymes. Direct influence of miR-17* on their regulation was demonstrated following miR-17* transfection [73]. Epithelial ovarian carcinoma exhibits decreased mi146a expression, and enforced expression of this miRNA results in a significant decrease in SOD2 expression and increased sensitivity to paclitaxel treatment [131]. Thus, many miRNAs have the potential to participate in the regulation of SOD2 mRNA stability and protein translation, and it is likely that many more miRNAs involved in SOD2 regulation await discovery. Delineating the context-dependent association between relative expressions of individual miRNA in SOD2 regulation requires more work.

## 6. Post-Translational Regulation of *SOD2*

**Figure 4:**
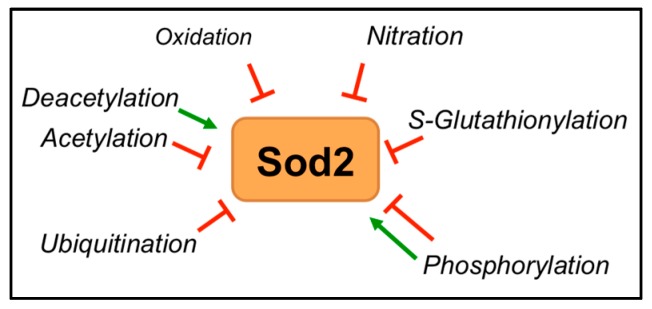
SOD2 post-translational regulation. Most modifications, besides phosphorylation, render the enzyme inactive. De-acetylation by SIRT enzymes is a common regulation of SOD2 activity in response to redox and nutrient stress.

Even though large-scale patient expression datasets, like The Cancer Genome Atlas (TCGA), give us important insights into the expression changes of SOD2 in different cancer types, these do not necessarily directly reflect on the activity of the enzyme. Since SOD2 can be regulated at the protein level by several post-translational modifications (Figure 4), there is the potential that its activity may be further regulated in the context of cancer. Moreover, it has been shown that increases in SOD2 expression do not always relate to reciprocal increases in SOD2 activity, as has been demonstrated in Alzheimer’s disease [132]. Another example is the expression of thioredoxin 1 (TRX1) in high-grade prostate cancers. Even though levels are significantly increased compared to low-grade specimens, TRX1 activity is inhibited due to TRX1 oxidation and enzyme inactivation [133]. As demonstrated below, post-translational modifications play a role in regulating SOD2 activity. However, unlike *SOD2* transcription, less is known about the regulation of SOD2 activity by these modifications in cancer.

### 6.1. Localization and Protein Interaction

SOD2 is a nuclear transcribed protein, which has an N-terminal mitochondrial leader sequence that targets its entry into the mitochondrial matrix. Here, it gets converted into its active form, following homotetramer assembly and Mn incorporation [134,135,136]. Thus, any variation in its amino acid sequence could result in protein conformational changes affecting its localization and function. SOD2 polymorphisms, including isoleucine-to-threonine substitution at amino acid 58 (Ile58Thr), which destabilized tetramer formation, and leucine-to-phenylalanine at codon 60 (Leu60Phe), result in decreased SOD2 activity and have consequences on cancer malignancy [136,137,138,139]. The most widely-characterized mutation however is a cytosine-to-thymine single nucleotide polymorphism (SNP), which causes substitution of alanine with valine at amino acid 16 (Ala16Val) of the mitochondrial target sequence in the pre-mature SOD2 protein. Proper recognition of this signal sequence by mitochondrial import factors located on the outer (translocase of outer membrane, TOM) and inner membranes (translocase of inner membrane, TIM23) is important for optimal matrix localization of SOD2 [140,141]. In addition, this SNP can cause inefficient cleavage of the signal peptide by Mitochondrial processing peptidase (MPP), resulting in protein mis-folding [136,142]. Moreover, the Ala16Val substitution has been reported to lead to 30–40% less dismutase activity compared to wild-type SOD2 [134,141,143]. This variant has also been shown to be associated with a higher risk of pancreatic and non-small-cell lung carcinoma [62,72]. Contrary to this, other studies have demonstrated that the wild-type alanine phenotype carries a greater risk of prostrate, ovarian and breast cancer, suggesting a pro-tumorigenic role for fully-functional SOD2 in these cancers [144,145,146]. Other mechanisms that could influence mis-compartmentalization of SOD2, but that have been largely unexplored in cancer, are abnormal cleavage of target sequences due to changes in mitochondrial MPP expression, protein mis-folding or the existence of dysfunctional mitochondria and mitochondrial protein import machinery, which can occur due to redox stress [147]. As discussed in Section 4.3, p53 localization to the mitochondria can negatively influence SOD2 activity by directly binding the protein [94], which is required for apoptosis initiation by p53. Interestingly, Sinclair et al., reported mis-localization of SOD2 to the cytosol and interaction with cytosolic caspases following prion infection in mice neuronal stem cells, leading to increased oxidative stress in the mitochondria and cell death [148]. Whether or not tumor cells are able to circumvent this mode of SOD2 inactivation, as a mechanism for apoptosis resistance, has not been explored. 

### 6.2. Transition Metal Incorporation

Each SOD2 subunit binds to a single Mn ion within its catalytic active site. In bacteria, the Mn incorporating enzyme is SOD A, which is closely related in overall primary sequence and structure to bacterial SOD B, a dismutase that utilizes Fe. Despite the striking sequence similarities, SODs are admirably metal specific. After SOD2 translocates to the mitochondrial matrix, its pre-sequence is cleaved, and subsequently, Mn incorporates into the enzyme making it fully functional [6,7]. However, finding a correct cofactor among a sea of diverse metals in a cell is a paramount task, and in vitro studies and work in yeast and *Escherichia coli* (*E. coli*) have demonstrated that manganese SODs can mistakenly bind Fe [6,149,150,151,152,153], resulting in their inactivation [154,155]. It is unclear if an imbalance in the Mn to Fe ratio in the mitochondrial matrix is a contributing factor to the regulation of SOD2 activity in cancer; however, it is well known that Fe imbalance and altered expression of iron transport proteins, such as the transferrin receptor, is a phenotype of cancer cells and can lead to changes in redox homeostasis [156]. siRNA-mediated silencing of the ABCB7 transporter in HeLa cells resulted in mitochondrial accretion of iron and SOD2 inactivation [157], suggesting that an altered Fe/Mn ratio in cancer cells could play a role in SOD2 activity regulation. 

In addition to a decrease in superoxide dismutase activity, Fe incorporation into *E. coli* manganese SOD A was shown to result in deleterious peroxidase activity of the enzyme [158]. Interestingly, high expression of SOD2 was shown to lead to redox damage of mitochondria, with the authors demonstrating a potential role for SOD2 peroxidase activity in this context [159]. However, it is not known if differential metal incorporation plays a role here. Fe-incorporating SOD2 or SOD A is also thermally more stable than Mn-SOD2; thus, making the situation more grim for abnormalities where iron overload occurs in the mitochondria [6]. 

### 6.3. Post-Translational Modifications

Protein function is dependent on its expression level and activity. Protein expression is, however, not always an estimate of its functional activity, which profoundly depends on the post-translational modifications that also regulate protein stability. In the next section, we will describe the post-translational modifications of SOD2 that have been explored in the context of cancers, leading to an imbalance in SOD2 protein or activity and consequential changes in mitochondrial redox homeostasis. 

#### 6.3.1. Acetylation

SOD2 undergoes active acetylation and deacetylation, making it functionally inactive or active, respectively [160]. Sirtuin 3 (SIRT3), a mitochondrial histone deacetylase, is the only known enzyme that deacetylates SOD2 [161,162,163]. SIRT3 activity is dependent on NAD^+^, and it is therefore an exquisite sensor of nutrient and redox stress, which leads to decreases in NAD^+^/NADH ratios. Several lysine acetylation sites have been reported on SOD2 including lysine (Lys) 122, 68, 53 and 89 residues [161,164,165]. SOD2-Lys68 acetylation also affects the mitochondrial p53-SOD2 interaction described above, which may play a role in nuclear-mitochondrial communication [165]. Another important acetylation site is Lys122 residue, which, under normal conditions, is acetylated and associated with basal SOD2 activity; however, upon SIRT3-mediated deacetylation, SOD2 activity is further increased [161]. Low SIRT3 expression and subsequent hyperacetylation and inactivation of SOD2 are thought to play a major role in the O_2_•^−^-driven activation of HIF-1α [166,167], leading to increased glycolysis in tumor cells [168]. In addition, knock-out animal studies demonstrate a tumor suppressor function for SIRT3 [160]. Loss of SIRT3 activity and hyperacetylation of SOD2 have been reported in many cancers including breast cancer [47,169], hepatocellular carcinoma [60], glioma [76] and B cell malignancies [63]. For example, mitochondrial calcium (Ca^2+^) has recently been implicated in suppressing SIRT3 activity in hepatocarcinoma through decreases in the NAD^+^/NADH ratio [60]. However, like SOD2, a dichotomous role for SIRT3 is emerging [170]. For example, it has been demonstrated that increased SIRT3 expression is found in cancer such as oral squamous cell carcinoma [171] and melanoma [172], suggesting that this could lead to concomitant increases in SOD2 activity. Moreover, the reliance of SIRT3 on the cofactor NAD^+^ has the potential to regulate SOD2 activity in response to low glucose conditions and redox stress, which occur during metastatic progression, such as anchorage independence [105]. Interestingly, SIRT3 is also under the regulation of Nrf2 [173]. Whether this represents an additional mechanism for SIRT3-mediated SOD2 activation in response to stress associated with tumor progression requires further investigation.

#### 6.3.2. Phosphorylation

The most common type of covalent modification is serine (Ser)/threonine/tyrosine phosphorylation, which is carried out by a diverse group of kinases within the cell and regulates the activity of most proteins. SOD2 is also a target of phosphorylation [6]. Using phospho-proteome analysis, Hopper et al., reported phosphorylated SOD2 in isolated mitochondria from pig heart, which resulted in SOD2 activity downregulation; while a two-fold increase in enzymatic activity was observed upon Ca^2+^-induced de-phosphorylation [174]. This suggests that Ca^2+^-dependent signaling processes may regulate SOD2 activity to modulate superoxide and hydrogen peroxide levels in the cell. In another study that investigated the effect of environmental low dose ionizing radiation, researchers showed that mitochondria-localized cyclin dependent kinase 4 (CDK4) directly phosphorylated SOD2 at Ser106, enhancing its activity and mitochondrial respiration [175]. CDK1 was similarly shown to increase Ser106 phosphorylation in response to radiation and thought to be an important stress adaptation [175,176]. Given that CDK4 is an important cell cycle regulator of cancer cells, this co-regulation of SOD2 requires further investigation in the context of tumor cells and their response to radiation and chemotherapy induced stress. In addition, it is unknown how other phosphorylation sites, such as Ser82, are involved in SOD2 regulation [177]. Phosphorylation sites in cytoplasmic SOD2 in *Listeria monocytogenes* [178] and mitochondrial SOD2 in potato [179] have been detected. In the bacteria, SOD A phosphorylation resulted in decreased enzymatic activity [178]. Taken together, SOD2 phosphorylation seems to be a conserved phenomenon from bacteria to vertebrates. Although not much has been reported about the effect of phosphorylation on SOD2 activity, this rapid covalent modification could be a possible mechanism of modulating highly dynamic signaling events involving ROS. Thus, further investigations need to be carried out to identify SOD2-specific kinases and phosphatases, SOD2 phosphorylation sites and the corresponding signaling pathways that regulate SOD2 phosphorylation in cancer.

#### 6.3.3. Oxidation, Nitration and S-Glutathionylation

Oxidative modifications can be associated with altering protein function, which can be reversible or irreversible. For example, reversible cysteine oxidation is a common mechanism for phosphatase inactivation. Protein hyperoxidation often leads to functional deficit of target proteins [180]. A recent mass spectrometry analysis showed that SOD2 oxidation at amino acid residues histidine (His54 and His55), tyrosine (Tyr58), tryptophan (Trp147, Trp149, Trp205 and Trp210) and asparagine (Asn206 and Asn209) resulted in impairment of enzyme activity in human kidney tissues [75]. Moreover, it was demonstrated that renal carcinoma cells display enhanced SOD2 oxidation at histidine (His54, His55), tyrosine (Tyr58) and tryptophan (Trp147, Trp149) residues [75], and human medulloblastoma cells have been previously shown to harbor hotspots for histidine (His54, His55) oxidation and SOD2 inactivation [48]. 

Nitration is a recognized modification of SOD2 [6] that was initially reported in a human renal allograft rejection model. While SOD2 protein levels were clearly detected, SOD2 enzymatic activity was decreased due to enhanced nitration [181]. The SOD2 catalytic site contains some highly conserved amino acid residues, and substitution of these residues results in either partial or complete loss of its enzymatic activity. Of importance is the highly conserved tyrosine residue (Tyr34), which is a target for peroxynitrite. Incorporation of 3-nitrotyrosine results in abrogation of SOD2 catalytic activity [6,34,181,182,183,184]. We found a single cancer-related study where Mallery et al., showed nitration and inactivation of SOD2 in acquired immune deficiency syndrome (AIDS)-related Kaposi’s sarcoma [59]. Thus, further investigation needs to be carried out to unfurl the existence and function of nitrated SOD2 in cancer. 

S-glutathionylation is the addition of glutathione at the cysteine residues of proteins and occurs during both stressed and unstressed conditions. It prevents irreversible oxidation of protein thiols and regulates diverse cellular processes [185]. In 2006, Hopper et al., reported S-glutathionylation of recombinant rat SOD2 grown in *E. coli*, but no functional studies were performed [174]. Cysteine (Cys) 196 was identified to be a target of glutathionylation in rat SOD2 [177]. In a more recent study, treatment with S-nitrosoglutathione significantly reduced SOD2 activity in a reversible manner in isolated rat kidneys [186]. However, more studies need to be performed to identify the role of this modification, especially in relevance to cancer, since it is associated with high oxidative stress.

#### 6.3.4. Ubiquitination

Protein turn-over is a highly regulated process carried out by the proteasomal degradation pathway where several ubiquitin ligases and deubiquitinating enzymes play a vital role. A study in coronary endothelial cells isolated from type 2 diabetic mice showed augmented SOD2 ubiquitination resulting in ROS accumulation and attenuation of coronary vascular relaxation [187]. Kim et al., demonstrated that a specific deubiquitinating enzyme, Ubiquitin Specific Peptidase 36 (USP36), extends the half-life of SOD2 by removing Ub groups and thereby increasing protein stabilization. A single report exists showing overexpression of the USP36 gene in ovarian cancer [188]. However, so far, no study has demonstrated a specific role of ubiquitinated SOD2 in cancer. 

## 7. Conclusions

From the above studies, it is clear that aberrant regulation of SOD2 expression and activity occurs throughout cancer development and progression. We believe that the dichotomous regulation and role of SOD2 can explain its role as both a tumor suppressor in early tumorigenesis and as a tumor promoter during metastatic progression. Given that the *SOD2* gene is rarely lost or mutated in cancer points to the fact that SOD2 regulation is adaptable. *SOD2* regulation by stress response pathways, such as NF-κB, hence allows for transient SOD2 expression in response to changing tumor microenvironments. For example, increased oxidative stress may activate SOD2 expression specifically in response to matrix detachment [24,105], or cytokines in the metastatic niche, such as TNF-α, may drive SOD2 expression to ensure survival and migration in this new environment [189]. The observations that cytokines and stress response transcription factors have a predominant role in SOD2 regulation imply that signals from the tumor microenvironment, produced in either an autocrine manner by tumor cells or in a paracrine fashion by tumor-associated macrophages and fibroblasts, may contribute to SOD2 regulation. However, few studies have directly assessed the role of tumor-associated cells on tumor cell SOD2 expression (e.g., co-culture models). It should be noted that studies have also pointed to altered SOD2 expression within tumor-associated cells themselves. For example, elevated SOD2 expression was identified as a major protein change in fibroblasts associated with ovarian cancer cells [190]. Given that expression data from large-scale datasets, including TCGA, contain a mixture of cancer cells and tumor-associated cells, the role and regulation of SOD2 between tumor cells and the tumor microenvironment need to be further delineated. 

While the transcriptional regulation leading to differential SOD2 expression has been the focus of much research, it is clear that the mechanisms of SOD2 post-translational regulation are less clearly delineated in cancer. This may be of particular importance when tumor cells require rapid adaptation to changing tumor environments. For example, immediate activation of SOD2 may be required as cells detach from the primary tumor, when matrix detachment initiates rapid redox stress and nutrient deprivation. Understanding the mechanisms of tumor cell adaptability to dynamically alter their antioxidant enzyme expression and activity will allow us to better target these for therapeutic intervention. While antioxidant therapy is clearly an undesirable approach in metastatic disease, pro-oxidant intervention with the combination of targeting the mechanisms that regulate antioxidants may be a beneficial strategy for tumor types that display enhanced SOD2 activity. 

## Figures and Tables

**Table 1:** Examples of regulatory mechanisms leading to dichotomous SOD2 expression and activity in cancer.

Tumor Type	Decrease	Mechanism	Increase	Mechanism
Bladder	↓Expression (Oncomine) ^1^	N/D ^2^		
Brain/CNS	↓Expression [36,37]	N/D	↑Expression [38,39,40,41,42,43,44,45,46] (Oncomine)	N/D
	↓Activity [47]	↓SIRT3 ^3^: SOD2 hyperacetylation [47]		
		oxidation [48]		
Breast	↓Expression (Oncomine)	p53 transcriptional inhibition (tumor initiation) [22];	↑Expression [22] (Oncomine)	Loss of p53 (tumor progression) [22];
		DDB2 ^4^ [49]		NF-κB ^8^ [50];
		Epigenetics [51,52]		Nrf2 ^9^ [53]
	↓Activity	↓SIRT3: SOD2 hyperacetylation [54]	↑Activity [55]	N/D
Colorectal	↓Expression [56]	Increased AP-1 ^5^ occupancy at promoter SNP ^6^ [56]	↑Expression [20,57] (Oncomine)	N/D
Esophageal	↓Expression [58] (Oncomine)	N/D	↑Expression (Oncomine)	N/D
Leukemia	↓Expression (Oncomine)		↑Expression	ARNT ^10^
Liver			↑Expression (Oncomine)	N/D
	↓Activity [59]	Ca^2+^ inhibition of SIRT3 [60]		
Lung			↑Expression [20,61]	N/D
	↓Activity	Ala16Val [62]		
Lymphoma			↑Expression (Oncomine)	N/D
	↓Activity	↓SIRT3: SOD2 hyperacetylation [63]		
Melanoma	↓Expression	LOH ^7^ [64]		
Multiple Myeloma	↓Expression	Epigenetic silencing [65,66]		
Ovarian			↑Expression [27,67] (Oncomine)	Keap1 mutation/Nrf2 activation [68]
Pancreatic	↓Expression	Epigenetic silencing [69]; miR-301a [70]	↑Expression [71] (Oncomine)	N/D
	↓Activity	Ala16Val [72]		
Prostate			↑Expression [20]	Low miR-17* expression [73]
Renal Clear Cell	↓Expression	HIF-1α [74]		
	↓Activity	Oxidation [75]		
Sarcoma	↓Expression (Oncomine)	N/D	↑Expression (Oncomine)	N/D
	↓Activity	Nitration [76]		
Tongue Squamous Cell	↓Expression	miR-222 [77]	↑Expression	c-myc ^16^ [78]

^1^ Oncomine [79] analyzed datasets that show changes in SOD2 expression relative to normal tissue controls; ^2^ N/D: mechanisms of regulation not determined; ^3^ SIRT3: sirtuin 3; ^4^ DDB2: damaged DNA binding 2; ^5^ AP-1: activating protein; ^6^ SNP: single nucleotide polymorphism; ^7^ LOH: loss of heterozygosity; ^8^ NF-κb: nuclear factor κB; ^9^ Nrf2: Nuclear Factor, Erythroid 2 Like 2; ^10^ ARNT: aryl hydrocarbon receptor nuclear translocator; ↑: increased; ↓: decreased.
